# Development of a simple measurement method for GluR2 protein expression as an index of neuronal vulnerability

**DOI:** 10.1016/j.toxrep.2014.12.014

**Published:** 2015-01-14

**Authors:** Chihiro Sugiyama, Yaichiro Kotake, Masafumi Yamaguchi, Kanae Umeda, Yumi Tsuyama, Seigo Sanoh, Katsuhiro Okuda, Shigeru Ohta

**Affiliations:** a Graduate School of Biomedical and Health Sciences, Hiroshima University, 1-2-3, Kasumi, Minami-ku, Hiroshima 734-8553, Japan; b Faculty of Pharmaceutical Sciences, Hiroshima International University, 5-1-1 Hirokoshinkai, Kure, Hiroshima 737-0112, Japan

**Keywords:** AMPA receptor, alpha-amino-3-hydroxy-5-methyl-4-isoxazole propionic acid receptor, DMEM, Dulbecco's modified Eagle's medium, DMSO, dimethyl sulfoxide, EDTA, ethylenediaminetetraacetic acid, FCS, fetal calf serum, Glu, glutamate, HS, horse serum, MAP2, microtubule-associated protein 2, NAS, 1-naphthylacetylspermine, PBS, phosphate-buffered saline, TBT, tributyltin, WST-1, 2-(4-iodophenyl)-3-(4-nitrophenyl)-5-(2,4-disulfophenyl)-2H-tetrazolium, GluR2, AMPA receptor, Neurotoxicity, AlphaLISA, Cell-based assay, Nitenpyram

## Abstract

*In vitro* estimating strategies for potential neurotoxicity are required to screen multiple substances. In a previous study, we showed that exposure to low-concentrations of some chemicals, such as organotin, decreased the expression of GluR2 protein, which is a subunit of alpha-amino-3-hydroxy-5-methyl-4-isoxazole propionic acid (AMPA)-type glutamate receptors, and led to neuronal vulnerability. This result suggested that GluR2 decreases as an index of neuronal cell sensitivity and vulnerability to various toxic insults. Accordingly, we developed a versatile method that is a large scale determination of GluR2 protein expression in the presence of environmental chemicals by means of AlphaLISA technology. Various analytical conditions were optimized, and then GluR2 protein amount was measured by the method using AlphaLISA. The GluR2 amounts were strongly correlated with that of measured by western blotting, which is currently used to determine GluR2 expression. An ideal standard curve could be written with the authentic GluR2 protein from 0 ng to 100 ng. Subsequently, twenty environmental chemicals were screened and nitenpyram was identified as a chemical which lead to decrease in GluR2 protein expression. This assay may provide a tool for detecting neurotoxic chemicals according to decreases in GluR2 protein expression.

## 1 Introduction

Mammals have been chronically exposed to environmental chemicals at low concentrations, and some environmental chemicals induce toxicity in individuals and in ecological systems. Thus, investigations of toxic environmental chemicals are necessary to prevent exposure. The central nervous system plays key roles in neuropsychiatric functions, such as behavior, learning, and memory, and comprises neuronal cells that recover poorly from damage. Life-long exposures to neurotoxic chemicals reportedly leads to altered behavior, mental retardation, and other neuronal disabilities, as well as diseases [1], [2], [3]. Moreover, during developmental stages, the immature blood–brain barrier allows the passage of neurotoxic environmental chemicals, even at low concentrations [4], [5]. Therefore, an index for neurotoxicity and a screening system for the index are required.

We previously showed that long-term exposure of rat cortical neurons to low concentrations of organotin decreases GluR2 protein expression, leading to increased neuronal susceptibility to glutamate stimulation compared with that in control neurons [6]. Subsequently, we showed that long-term lead exposure induces neuronal cell death in association with decreased GluR2 expression [7]. The GluR2 protein is a subunit of the alpha-amino-3-hydroxy-5-methyl-4-isoxazole propionic acid (AMPA) receptor, which is a glutamate receptor that mediates rapid excitatory synaptic transmissions in the central nervous system. GluR2 is critical for Ca^2+^ permeability of AMPA receptors. GluR2-containing AMPA receptors are impermeable to Ca^2+^ [8], whereas GluR2-lacking AMPA receptors are highly permeable to Ca^2+^ in a steady state, and the majority of functional AMPA receptors contain GluR2 [9]. GluR2 plays important roles in neuronal death, such as that associated with ischemia or Alzheimer's disease [10], [11], [12]. In addition, recent studies show that GluR2 is an essential regulator of the memory phenomena [13]. These studies suggest that decreases in GluR2 may be utilized as an index for conditions under which neuronal cells are sensitive and vulnerable to other stimulants. To investigate multiple neurotoxic chemicals for their effects on GluR2 expression, we need large-scale determinations of GluR2 protein expression in neuronal cells. However, western blotting for GluR2 expression is unsuitable for high throughput screening.

AlphaLISA^®^ (developed by PerkinElmer, Inc.) is applied to high throughput screening methods with high reproducibility [14], [15], [16]. This analysis is a bead-based proximity immunoassay [17] that exploits oxygen channeling technology. AlphaLISA assays are performed with anti-analyte (a target protein) antibodies and two AlphaLISA beads, including streptavidin-coated alpha donor beads and IgG-coated alpha acceptor beads. AlphaLISA is an all-in-one-well assay that does not require transfer or wash steps, and allows assessments of analytes on a large scale [18].

**Fig. 1:**
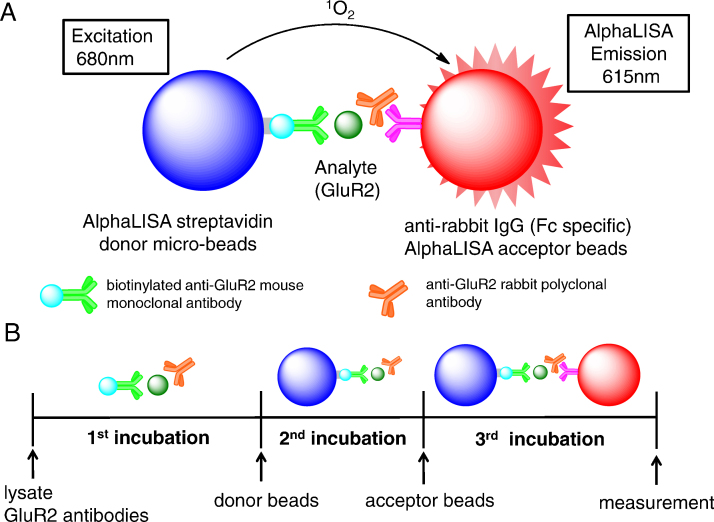
AlphaLISA assay of GluR2 protein expression. (A) Principles of the AlphaLISA assay; biotinylated anti-GluR2 mouse monoclonal antibody binds streptavidin-coated alpha donor beads and anti-GluR2 rabbit polyclonal antibody binds anti-rabbit IgG-coated alpha acceptor beads. Both antibodies recognize GluR2, allowing the beads to come into close proximity. The excitation of the donor beads at 680 nm provokes the release of singlet oxygen molecules that trigger an energy transfer cascade in acceptor beads, resulting in a sharp emission peak at 615 nm. (B) AlphaLISA assay protocol.

In this study, we developed a novel *in vitro* assay to screen multiple compounds for their effects on GluR2 expression by means of AlphaLISA technology (Fig. 1). Subsequently, twenty potentially neurotoxic chemicals were screened, and then nitenpyram was identified as a novel chemical which lead to decrease in GluR2 protein expression.

## 2 Materials and methods

### 2.1 Materials

Eagle's minimal essential salt medium (Eagle's MEM) was purchased from Nissui Pharmaceutical (Tokyo, Japan). Fetal calf serum (FCS) was purchased from Nichirei Biosciences (Tokyo, Japan). Horse serum (HS) was purchased from Gibco (Life Technologies, Carlsbad, CA, USA). Acifluorfen, bithionol, bromofenofos, carbofuran, *cis*-permethirin, d-(+)-glucose, 4,4′-dichlorobenzophenone, dertamethrin, diclofop, dimethyl sulfoxide (DMSO), fensulfothion, fenthion, methiocarb, NaHCO_3_, nitenpyram, nitrixynil, ortho phenyl phenol, oxyclozanid, phenylmethylsulfonyl fluoride (PMSF), procloraz, rafoxianid, Sodium dodecyl sulfate (SDS), tribromsalan and 2-(4-iodophenyl)-3-(4-nitrophenyl)-5-(2,4-disulfophenyl)-2H-tetrazolium (WST-1) were purchased from Wako (Tokyo, Japan). Allethrin, arabinosylcytosine, anti-β-actin antibody (AC-15) and 1-naphthylacetylspermine (NAS) were purchased from Sigma–Aldrich (St. Louis, MO, USA). Pentobarbital was purchased from Kyoritsu (Tokyo, Japan). Captafol and dichlorvos were purchased from Kanto Chemical Co., Inc. (Tokyo, Japan). Tris–HCl, nonidet P-40, ethylenediaminetetraacetic acid (EDTA), mercaptoethanol, and protease inhibitor cocktail were purchased from Nacalai Tesque (Kyoto, Japan). pGEX-6P and PreScission protease were purchased from GE healthcare (Piscataway, NJ). BL-21-CodonPlus (DE3) RIPL was purchased from Agilent Technologies (Santa Clara, CA). Mouse anti-GluR2 monoclonal antibody (MAB397) was purchased from Millipore (Billerica, MA, USA). Rabbit anti-GluR2 pAb (BS3658) was purchased from Bioworld Technology, Inc. (St. Louis, MN, USA). ChromaLink™ Biotin (DMF Soluble) was purchased from solulink (San Diego, CA, USA). Zeba Desalt Spin Columns (0.5 mL) and Pierce™ BCA Protein Assay Kits were purchased from Thermo Fisher Scientific K.K. (Rockford, IL, USA). Bromoxynil was purchased from Dr. Ehrenstorfer GmbH (Augsburg, Germany).

AlphaLISA streptavidin donor micro-beads (676002S), anti-rabbit IgG (Fc specific) AlphaLISA acceptor beads (AL104C), AlphaLISA immunoassay buffer (AL000C), and 1/2 AreaPlate-96 plates were purchased from PerkinElmer, Inc. (Waltham, MA02451, USA).

### 2.2 Cell culture

The present study was approved by the animal ethics committee of Hiroshima University. The following procedures were performed under sterile conditions. Primary cultures were obtained from the cerebral cortex of fetal rats (at 18 days of gestation) as described previously [19]. Fetuses were taken from pregnant Slc; Wistar/ST rats under pentobarbital anesthesia. Subsequently, parts of the cerebral cortex were dissected using a razor blade, and cells were dissociated by gentle pipetting and plated on culture plates (4 × 10^6^ cells/cm^2^). Cultures were incubated in Eagle's MEM supplemented with 10% fetal calf serum, l-glutamine (2 mM), d-(+)-glucose (11 mM), NaHCO_3_ (24 mM), and HEPES (10 mM). Cultures were maintained at 37 °C in a humidified 5% CO_2_ atmosphere. Cultures were incubated in MEM containing 10% FCS (1–7 days) or 10% HS (8–11 days) and the medium was exchanged every 2–3 days. Arabinosylcytosine (10 μM) was added to inhibit the proliferation of non-neuronal cells at 6 days *in vitro* (DIV). Cultures were used for experiments at 11 DIV. This protocol has been shown to produce cultures containing approximately 90% neurons, as indicated by immunostaining for the neuron marker, microtubule-associated protein 2 (MAP2).

### 2.3 Sample preparation

Cultures were used at 11 DIV. After chemical treatment, cells were washed with PBS and lysed in TNE buffer containing 50 mM Tris–HCl, 1% nonidet P-40, 20 mM EDTA, Protease Inhibitor Cocktail (1:200), and 1 mM PMSF. Mixtures were mixed by rotation at 4 °C and were centrifuged at 15,000 rpm; supernatants were used as cell lysates for AlphaLISA assays and western blotting.

### 2.4 Biotinylation and desalting of anti-GluR2 antibodies

Two kinds of anti-GluR2 antibodies with differing recognition sites on the GluR2 protein were selected for AlphaLISA, including mouse anti-GluR2 monoclonal antibody, which recognizes 175–430 amino acids of GluR2 protein, and rabbit anti-GluR2 polyclonal antibody, which recognizes 850–880 amino acids of GluR2 protein. Biotinylation of mouse anti-GluR2 monoclonal antibody was performed using ChromaLink™ Biotin (DMF Soluble; Solulink). Briefly, 100 μl of 1 mg/ml antibody, 7.6 μl of 2 mg/ml NHS-ChromaLink-biotin reagent, and 92.4 μl of PBS were incubated for 2 h at 23 °C. The biotinylated antibody was purified using a Zeba Desalt Spin Column of 0.5 mL (Thermo Fisher Scientific K.K) according to the manufacturer's protocol. After biotinylation, absorbances at 280 nm (protein), 354 nm (biotin), and 450 nm (turbidity) were determined using a Multiskan™ GO (Thermo Fisher Scientific K.K). The number of biotins per antibody was calculated using a ChromaLink Biotin E1% MSR Calculator according to absorbance values. Desalting of rabbit anti-GluR2 polyclonal antibody was also performed using a Zeba Desalt Spin Column of 0.5 mL.

### 2.5 AlphaLISA assay

The AlphaLISA assay was performed in a final volume of 50 μl containing cell lysates, biotinylated mouse anti-GluR2 monoclonal antibody, rabbit anti-GluR2 polyclonal antibody, streptavidin-coated donor beads, and anti-rabbit IgG AlphaLISA acceptor beads. Cell lysates were prepared in TNE buffer and the other reagents were diluted with AlphaLISA immunoassay buffer. Dilute solutions were used as negative controls.

Cell lysate (5 μl) and two kinds of anti-GluR2 antibodies (in 10 μl) were added to 1/2 AreaPlate-96 culture plates (PerkinElmer, Inc.) and were incubated at 23 °C (1st incubation). Subsequently, 20 ng/mL streptavidin-coated donor beads (12.5 μl) were added and incubated (2nd incubation), and then 20 ng/ml of acceptor beads (12.5 μl) were added and incubated (3rd incubation) in the dark. Measurements were performed using a multimode plate reader (Enspire™; PerkinElmer, Inc.).

Each examination was carried out on clamped conditions as follows:

• Determinations of antibody concentrations; 1st incubation time, 2 h; 3rd incubation time, 4 h; protein concentration, 3000 ng.
• Determinations of protein abundance; 1st incubation time, 2 h; 3rd incubation time, 4 h; biotinylated anti-GluR2 monoclonal antibody, 10 nM; anti-GluR2 polyclonal antibody, 5 nM.
• Assessments of incubation times; biotinylated anti-GluR2 monoclonal antibody, 10 nM; anti-GluR2 polyclonal antibody, 5 nM; protein concentration, 3000 ng.

### 2.6 Generation of GluR2 constructs

GluR2 constructs were generated using inverted PCR with the following oligonucleotides:

PRM1, TCTAGAATGCAAAAGATTATGCATATTTCTGTC;
PRM2, GCGGCCGCCTAAATTTTAACACTCTCGATGCCATATAC;
PRM3, AAGAAAGGAAAACACTCCTGGTTTG;
PRM4, GAGTTCTGTTACAAGTCAAGGGCC;
PRM5, GGATCCGAGAAGAAGTGGCAGGTGACTGCTATC.

GluR2Δ1–169, 539–834 fragments were constructed using a three-step PCR process. First, GluR2 gene fragments were amplified from rat brain cDNA using the primers PRM1 and PRM2, and amplified fragments were ligated into pBlueScript II. Second, the GluR2Δ539–834 gene was amplified from pBlueScript II/GluR2 using the primers PRM3 and PRM4. Third, the GluR2Δ1–169, 539–834 gene was amplified from pBlueScript II/GluR2Δ539–834 using the primers PRM5 and PRM2. The GluR2Δ1–169, 539–834 fragment was then digested using BamHI and NotI and was cloned into pGEX-6P.

### 2.7 GluR2Δ1–169, 539–834 protein expression

Expression of GST-tagged GluR2Δ1–169, 539–834 protein in *Escherichia coli* strain BL-21-CodonPlus (DE3) RIPL was induced using 0.1 mM IPTG at 37 °C for 1 h. Bacterial pellets were stored at −80 °C. Subsequently, bacterial pellets from 8-L cultures were thawed in extraction buffer containing 20 mM Tris–HCl (pH 8.0), 50 mM NaCl, 5 mM EDTA, 10 mM DTT, 0.1% Triton-X100, 1 mM PMSF, 2 μM leupeptin, and 2 μM pepstatin A, and were sonicated at mild intensity. After centrifugation for 30 min at 24,000 × *g*, extracts were rotated with 1 mL of glutathione sepharose at 4 °C for 2 h, and then sepharose was washed using digestion buffer containing 50 mM Tris–HCl (pH 7.0), 150 mM NaCl, 1 mM EDTA, 1 mM DTT, 0.1% Triton-X 100, and 1% glycerol. For enzyme digestion, sepharose was incubated overnight with 20 μL of PreScission protease at 4 °C. Digested GluR2Δ1–169, 539–834 protein was dialyzed against buffer containing 20 mM Tris–HCl (pH 7.0), 150 mM NaCl, 1 mM EDTA, and 1 mM DTT, and was used as a purified GluR2 standard.

### 2.8 Western blotting

Western blotting experiments were performed as described previously [20]. Briefly, supernatants were added to the sample buffer containing 100 mM Tris–HCl, 4% SDS, 20% glycerol, 0.004% bromophenol blue, and 5% mercaptoethanol, and were then denatured at 95 °C for 3 min. Proteins were separated using SDS-polyacrylamide gel electrophoresis and were transferred to polyvinylidene difluoride membranes. Membranes were blocked with a blocking buffer containing 5% skim milk for 1 h, and were then incubated with anti-GluR2 (1:2000) and anti-β-actin (1:8000) antibodies overnight at 4 °C. After incubation with secondary antibody for 1 h, proteins were detected using an enhanced chemiluminescence detection system [Chemi-Lumi One L, Nacalai Tesque (Kyoto, Japan)]. Quantitative analyses were performed using digital imaging software [Image J, NIH (Bethesda, MD, USA)], and GluR2 protein levels were normalized to those of β-actin. Western blotting was performed to confirm associations of anti-GluR2 antibody with either donor beads or acceptor beads and the subsequent formation of the complex for AlphaLISA assays.

### 2.9 Cell viability assay

Trypan blue dye exclusion assays and WST-1 assay were performed to assess neurotoxicity. Trypan blue dye exclusion assays were performed as described in previous reports [19]. After exposure to nitenpyram, cell cultures were immediately stained with 1.5% trypan blue for 10 min. Subsequently, the cells were fixed with 10% formalin for 2 min and rinsed with physiological saline. Unstained cells were considered viable and stained cells were considered dead. Cell viability of cultures was calculated as the percentage ratio of the number of unstained cells to total cells counted. Over 200 cells per well were randomly counted. In WST-1 assay, after exposure to nitenpyram, a mixed solution of WST-1, 1-methoxy-5-methylphenazinium methylsulfate, and DMEM was added to each well, and the plate was incubated for 1 h. Finally, the supernatants were transferred to the wells of a 96-well plate, and the absorption was measured at 415 nm, the maximum wavelength of the formazan dye, with a Microplate reader, Multiscan™ GO (Thermo Scientific).

### 2.10 Statistics

All the experiments were replicated and representative data were shown. Data are expressed as means ± standard errors of the mean (SEM). Statistical analyses were performed using ANOVA followed by Tukey's test, and differences were considered significant when *p* < 0.05.

## 3 Results

### 3.1 Complex formation for AlphaLISA assays

**Fig. 2:**
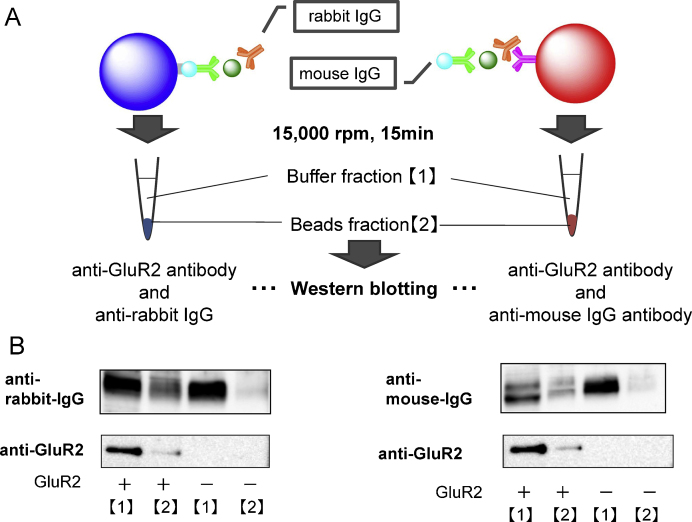
GluR2 complex formation for AlphaLISA assays. (A) Cell lysates, anti-GluR2 antibodies, and AlphaLISA beads were incubated in microtubes without anti-rabbit IgG-coated alpha acceptor beads or without streptavidin-coated alpha donor beads. After incubation, bead fractions were separated by centrifugation at 15,000 rpm for 15 min. (B) Western blotting of fraction [1] buffer with extra antibodies and extra GluR2; fraction [2] beads bound with antibodies and GluR2. TNE buffer with cell lysis buffer was used as a control. (B) Western blotting of fraction [1], buffer with extra antibodies and extra GluR2 fraction [2], beads bound with antibodies and GluR2. TNE buffer with cell lysis buffer was used as a control.

Because AlphaLISA is based on the sandwich configuration, associations of each anti-GluR2 antibody with either donor beads or acceptor beads, and formation of corresponding complexes, were confirmed by means of western blotting with AlphaLISA reaction mixtures (Fig. 2). As in the AlphaLISA protocol in Fig. 1B, cell lysates containing GluR2 protein, anti-GluR2 antibodies, and beads for AlphaLISA were added and incubated for 2 h. Subsequently, two types of mixtures were prepared by incubation without donor or acceptor beads and were then centrifugation at 15,000 rpm for 15 min to separate bead and buffer fractions. As a control, TNE buffer was added instead of cell lysates [GluR2(−)]. GluR2 protein and anti-GluR2 antibodies in each mixture were detected by means of western blotting. In samples without donor beads and in samples without acceptor beads, GluR2 and anti-GluR2 antibodies were concentrated in the precipitated beads fraction [GluR2(+)] compared with the fraction from control samples [GluR2(−); Fig. 2]. These data demonstrate the formation of essential complexes between the tested anti-GluR2 antibodies and beads, and validate their use in AlphaLISA assays of GluR2 in cell lysates.

### 3.2 Optimization of assay conditions

**Fig. 3:**
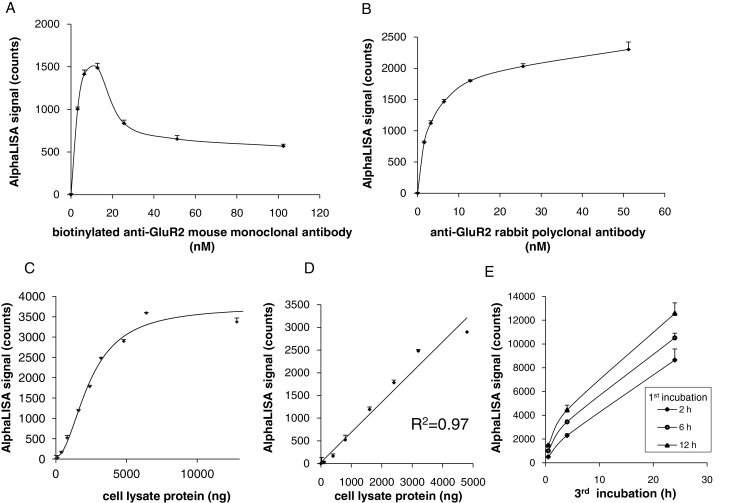
Optimized assay conditions for detecting GluR2 expression. (A) Determination of optimum concentrations of biotinylated anti-GluR2 mouse monoclonal antibody, and (B) anti-GluR2 rabbit polyclonal antibody; (C) determination of optimum protein concentrations of cell lysates; rat primary cerebral cortical neurons were lysed in TNE buffer, and total protein abundance was quantified using bicinchoninic acid (BCA) protein assays; (D) enlarged view of (C); (E) time course of incubation: 1st incubation, binding of GluR2 protein and anti-GluR2 antibodies; 3rd incubation, binding of acceptor beads and anti-GluR2 rabbit polyclonal antibody. Data are expressed as the mean ± standard error of the mean (*n* = 3).

All antibodies had bead-binding capacity and the peak AlphaLISA signals were observed before saturation (hook point). Optimal concentrations of each antibody were identified according to bell-shaped curves of biotinylated mouse anti-GluR2 monoclonal antibody signals (Fig. 3A). The peak signal was observed at 12.8 nM indicating the hook point. In subsequent optimization experiments, biotinylated anti-GluR2 monoclonal antibody was used at the sub-hooking concentration of 10 nM. A saturated curve was obtained for rabbit anti-GluR2 polyclonal antibody (Fig. 3B), which required coupling to anti-rabbit IgG antibodies on acceptor bead surfaces. Under these conditions, acceptor beads became saturated with anti-GluR2 antibody, which was used at 5 nM to avoid saturation in further experiments. Hook points were also found in AlphaLISA experiments with cell lysates from rat primary cerebral cortical neurons, allowing determination of optimum protein amount in cell lysates (Fig. 3C). An AlphaLISA saturation curve was obtained using lysates from unexposed rat primary cerebral cortical neurons and was linear up to about 5000 ng (*R*^2^ = 0.97; Fig. 3D). Thus, cell lysates with a protein content of 3000 ng were used in further experiments.

Reactions of both the 1st and the 3rd incubations of antibodies were enhanced with the duration of incubation. A time-dependent increase was also observed at both 1st and 3rd incubation times, and counts did not saturate (Fig. 3E). Therefore, for further experiments the 1st and the 3rd incubation times were set at 6 and 15 h, respectively.

### 3.3 Correlation between western blotting analyses and AlphaLISA assays

**Fig. 4:**
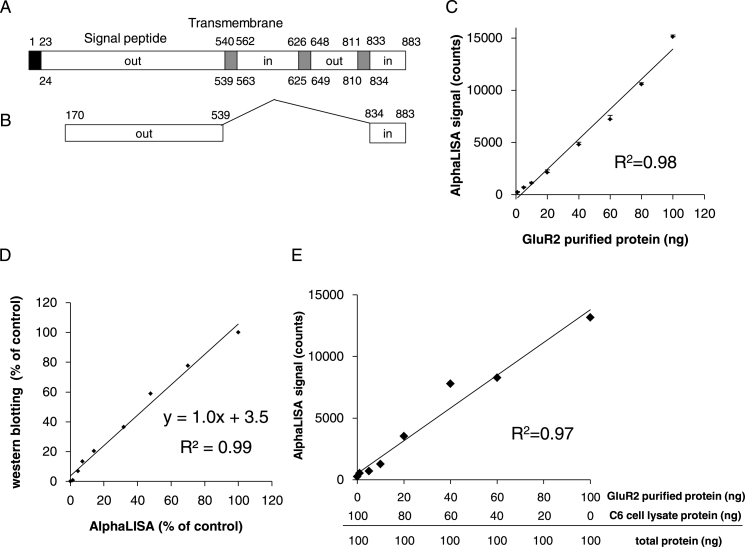
Schematic representation of GluR2 protein and AlphaLISA assay. (A) GluR2 is a predicted six-transmembrane protein; (B) Schematics of the GluR2Δ1–169, 539–834 construct; numbers above the amino acid residues refer to residues of the full-length receptor; (C) measurement of purified GluR2 standard using AlphaLISA. Data are expressed as the mean ± the standard error of the mean (*n* = 3). (D) Correlation between western blotting analyses and AlphaLISA assays. Quantitative western blotting analyses of GluR2 protein expression were performed using image J software. (E) Interference of the other proteins except GluR2 protein by means of the lysate of C6 cells. AlphaLISA was conducted using identical samples which contain the GluR2 standard and the lysates of C6 cells.

After optimizing assay conditions, in order to validate the present AlphaLISA assay, experiments were performed with a purified GluR2 standard. GluR2 is a predicted six-transmembrane protein (Fig. 4A) and we generated GluR2 constructs using inverted PCR (Fig. 4B). In these experiments AlphaLISA signals were linear up to 100 ng (*R*^2^ = 0.98; Fig. 4C) and were specific to the GluR2 protein. In addition, two kinds of the GluR2 determinations, AlphaLISA and western blotting, were conducted to the identical samples which contains 0–100 ng GluR2 standard, and the result showed strong correlation between the methods (*R*^2^ = 0.99, Fig. 4D). The correlation indicated that the present AlphaLISA assay can be used as an alternative for western blotting. Moreover, we investigated the interference of the other proteins except GluR2 protein by means of the lysate of C6 cells. We conducted to the identical samples which contain the GluR2 standard and the lysates of C6 cells. The result showed strong correlation between the methods (*R*^2^ = 0.97, Fig. 4E) in the presence of the protein from C6 cells and there is slightly difference compared with the samples which only contains GluR2 standard and TNE buffer (Fig. 4C).

### 3.4 Identification of nitenpyram as a novel suppressor of GluR2 expression

**Fig. 5:**
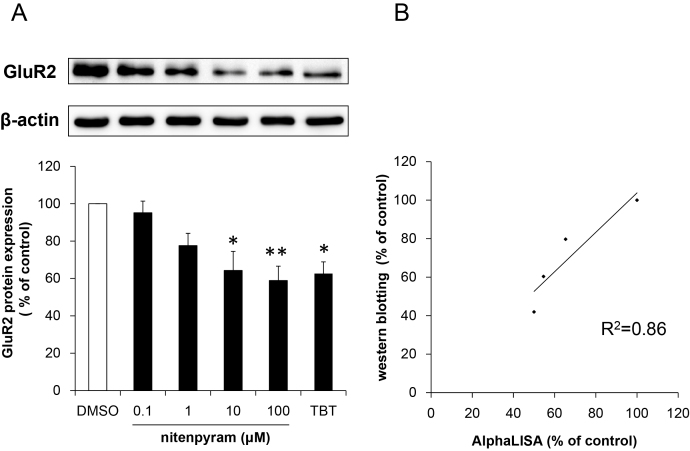
Changes in protein expression of GluR2 following long-term exposure to nitenpyram or TBT. (A) Cortical neurons were exposed to 0.1–100 μM NIT or 50 nM TBT for 9 days from 2 days *in vitro* (DIV) to 10 DIV, and GluR2 was then detected using western blotting. Quantitative analysis of GluR2 western blots were performed using ImageJ software, and GluR2 protein levels were normalized to those of β-actin. Data are expressed as the mean + standard error of the mean (*n* = 4) *p < 0.05, **p < 0.01 vs. DMSO. (B) Correlation between western blotting analyses and AlphaLISA assays. Quantitative western blotting analyses of GluR2 protein expression were performed using image J software.

Twenty environmental chemicals were screened for the effect on GluR2 expression by means of the present AlphaLISA method. We previously showed that long-term exposure of rat cortical neurons to environmental chemicals, organotin and lead, led to decreased GluR2 protein expression and greater susceptibility of neurons to glutamate stimulation [6], [7]. However, We think GluR2 decrease may be caused by other environmental chemicals besides metals, and screened the other type of environmental chemicals, such as agricultural chemicals. We selected twenty environmental chemicals which have various principle uses, such as pesticide, antiparasitic, herbicide and bactericide as test compounds for screening. Neuronal cells were exposed to DMSO (control) or test compounds for 9 days at the concentration of 1 μM and 10 μM (Table 1). As the results, the neonicotinoid insecticide nitenpyram decreased the most in GluR2 expression. In subsequent experiments, cortical neurons were exposed to 0.1–100 μM nitenpyram for 9 days and GluR2 expression was measured. As we previously reported that showed that long term exposure to 20 nM tributyltin (TBT) leads to decreased GluR2 protein expression in rat cortical neurons [6]. TBT-treated cortical neurons were used as a positive control, and GluR2 expression was reduced by nitenpyram in a dose-dependent manner and the reduction was significant at concentrations above 10 μM (Fig. 5A). AlphaLISA and western blotting were conducted to the identical samples which were obtained from neuronal cells which were exposed to 0–100 μM nitenpyram, and the result showed strong correlation between the methods (*R*^2^ = 0.86, Fig. 5B), which indicated that AlphaLISA assay is useful to assess the neurotoxicity of large numbers of hazardous chemicals as an alternative for western blotting.

**Table 1:** Test compounds for the screening of GluR2 decrease.

Principal use	Chemical	Mechanism of action	AlphaLISA (% of control)	
			1 μM	10 μM
Pesticide	Dichlorvos	Acetylcholinesterase inhibitor	107	89
	Fenshlfothion	Acetylcholinesterase inhibitor	118	111
	Fenthion	Acetylcholinesterase inhibitor	90	87
	Carbofuran	Acetylcholinesterase inhibitor	80	90
	4,4′-Dichlorobenzophenone	Sodium channel modulator	76	107
	Allethrin	Sodium channel agonist	97	84
	Dertamethrin	Sodium channel agonist	100	94
	*cis*-Permethirin	Sodium channel agonist	99	84
	Nitenpyram	Nicotinic acetylcholine receptor agonist	81	66

Antiparasitic	Rafoxianide	ATP syntesis inhibitor	126	88
	Bithionol	ATP syntesis inhibitor	98	71
	Oxyclozanide	Uncoupler of oxidative phosphorylation	103	104
	Bromofenofos	Uncoupler of oxidative phosphorylation	107	82
	Nitrixynil	Uncoupler of oxidative phosphorylation	101	99
	Tribromsalan	Uncoupler of oxidative phosphorylation	83	106

Herbicide	Diclofop	Acetyl-CoA carboxylase inhibitor	84	105
	Acifluorfen	Protoporphyrinogen oxidase inhibitor	82	79
	Bromoxynil	Inhibition of photosynthesis	96	96

Bactericide	Procloraz	Inhibition of sterol 14-demethylation	145	112
	Ortho phenyl phenol		119	93

### 3.5 Vulnerability of nitenpyram-treated cells to glutamate stimulation

**Fig. 6:**
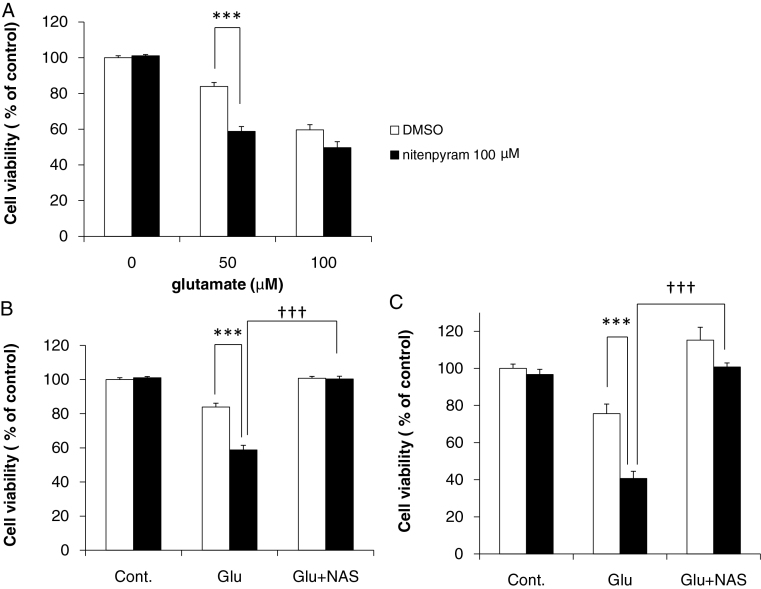
Influence of long-term exposure to nitenpyram on glutamate toxicity. (A) Cortical neurons were exposed to 50 and 100 μM glutamate for 24 h after 100 μM nitenpyram treatment. Data are expressed as the mean + standard error of the mean (*n* = 3); ***p < 0.001, ^†††^*p* < 0.001 vs. DMSO. (B) Glutamate (50 μM) was applied to neurons 30 min after treatment with 300 μM NAS and cell viability was measured by trypan blue assay. Data are expressed as the mean + standard error of the mean (*n* = 3). (C) Glutamate (50 μM) was applied to neurons 30 min after treatment with 300 μM NAS and cell viability was measured by WST-1 assay. Data are expressed as the mean + standard error of the mean (*n* = 6).

To determine whether nitenpyram causes cell vulnerability, we assessed glutamine-induced neuronal death in the presence or absence of nitenpyram. Neurons were exposed to DMSO (control) or 100 μM nitenpyram for 9 days, followed by 0, 50, or 100 μM glutamate for 24 h. At 50 μM concentration, cell viability following glutamate stimulation differed significantly between control neurons and nitenpyram-treated neurons (Fig. 6A). Moreover, this increased susceptibility of nitenpyram-treated neurons was abolished by an antagonist of GluR2-lacking AMPA receptor, 1-naphthylacetylspermine (NAS) (Fig. 6B and C).

## 4 Discussion

Some environmental chemicals are believed to have neurotoxicity at low concentrations [3]. However, small numbers of chemicals have been assessed for neurotoxicity as well-developed methods and markers of neurotoxicity are not available. Analyses of behavioral and neuropathological changes *in vivo* provide powerful tools for assessing neurotoxicity [21]. Since such analyses are time consuming and expensive, they are unsuitable for neurotoxicity screening of multiple chemicals, necessitating a convenient high throughput *in vitro* method [22], [23]. Thus, in this study, we conditioned AlphaLISA assay for the measurement of GluR2 which is known as a key subunit of AMPA receptor and dictate Ca^2+^ permeability. The assay was utilized to assess decreases in GluR2 expression following long-term exposure to low concentrations of multiple environmental chemicals.

AlphaLISA assays are performed with anti-analyte (a target protein) antibodies and two AlphaLISA beads, including streptavidin-coated alpha donor beads and IgG-coated alpha acceptor beads. In this method, biotinylated anti-analyte antibodies are conjugated to donor beads and non-biotinylated anti-analyte antibodies are conjugated to acceptor beads. Accordingly, the acceptor bead is not directly coupled to the analyte-specific antibody, but is coupled to another antibody that recognizes the non-biotinylated anti-analyte antibody. Upon excitation, a photosensitizer inside the donor bead converts ambient oxygen to an excited singlet state. In the presence of analytes, antibodies and beads conjugate analytes, and singlet oxygen molecules are provoked by donor beads that are proximal (about 200 nm) to acceptor beads, triggering a cascade of chemical events in nearby acceptor beads and chemiluminescent emissions at 615 nm.

Under optimal conditions, GluR2 protein amount in AlphaLISA assays and western blotting analyses were highly correlated (*R*^2^ = 0.99, Fig. 4D). The regression line is corresponding to the unity because the slope is about 1. Western blotting is used as a standard method for confirming the expression of specific proteins. However, it requires electrophoresis and transfer steps, followed by quantitation of band densities using software, and lacks quantitative accuracy. In contrast, AlphaLISA is an all-in-one-well assay that does not require transfer or wash steps and the signals were quantitative with a purified GluR2 standard (*R*^2^ = 0.98, Fig. 4C), confirming specificity for the GluR2 protein. Moreover, the strong correlation between AlphaLISA assays and western blotting analyses validates the present AlphaLISA method as a rapid alternative to western blotting for measurements of GluR2 expression. Besides we confirmed that AlphaLISA was not interfered other cell lysate proteins expect GluR2 using the samples which contain the GluR2 standard and the lysates of C6 cells (Fig. 4E). This results also suggest that TNE buffer is enough as a blank, not cell lysates which do not contain GluR2 protein.

After screening twenty environmental chemicals using the present method, nitenpyram, one of the neonicotinoid insecticides, was identified as a candidate that decreases GluR2 expression (Table 1). Nitenpyram treatment significantly increased neuron susceptibility to glutamate and a GluR2-lacking AMPA receptor antagonist rescued this susceptibility (Fig. 6), suggesting that nitenpyram-induced GluR2 decrease leads to susceptibility of neurons. These results validated the present AlphaLISA method for assessments of neurotoxicity.

Neonicotinoid insecticides are currently the newest and largest single insecticide class in the market, representing more than 20% of insecticides used in 2008 globally [24]. Neonicotinoid insecticides are also used to control fleas and ticks in household pets, and act as selective agonists of nicotinic acetylcholine receptors (nAChRs) in insects [25]. However, some reports suggest that neonicotinoid insecticides affect mammalian nAChRs to a greater extent than previously believed [26], [27], [28] and that further studies of neonicotinoid insecticides are required.

We developed a new *in vitro* evaluating method utilizing decreased GluR2 protein expression results in detectable neuronal cell vulnerability [6], [7], as well as automated image analyses of neurogenesis and apoptosis. GluR2 is a subunit that depresses cell permeability to bivalent cations in a steady state [29], [30] and plays an important role in glutamatergic neuron signaling. Previous studies have demonstrated that GluR2 decreases damage active neurons, and that GluR2 knockout mice have impaired associative learning and reduced anxiety [31]. In *in vitro* studies, neurotoxicity is evoked by kainate-acid stimulation in GluR2-deficient neurons, possibly reflecting other GluR2-associated consequences in addition to Ca^2+^ permeability [32]. Moreover, decreases in GluR2 protein expression have been related to neurodegenerative diseases [10], [11], [12]. Accordingly, about 60–70% of AMPA receptors have intracellular localization [33], but are delivered to the plasma membrane surfaces as functional AMPA receptors. Thus, total decreases in GluR2 reflect those on the plasma membrane surface.

We previously showed that long-term exposure of rat cortical neurons to environmental chemicals led to decreased GluR2 protein expression and greater susceptibility of neurons to glutamate stimulation [6], [7]. These data suggest that GluR2 decreases may offer an index of neuronal cell sensitivity and vulnerability to other stimuli. However, further studies are needed to confirm that GluR2 decreases are a suitable endpoint of neurotoxicity. To this end, comparisons of the effects of multiple chemicals on GluR2 protein expression using AlphaLISA, which is a high throughput screening method with high reproducibility, will be valuable. In the present study, we simplified the AlphaLISA procedures following cell solubilization. However, future studies are required to simplify the pre-measurement processing, such as sample preparation, to maximize the practical application of the present method. In addition, in the present method, AlphaLISA needs three steps for measurement. However, we can simply the method to two steps by binding the GluR2 antibody with GluR2 antibodies which are used in the present method to acceptor beads in advance (AlphaLISA direct assay). AlphaLISA direct assay is capable of improvement in both cost and time consumption. In conclusion, we developed a simple high throughput measurement method for GluR2 expression by means of AlphaLISA technology. This AlphaLISA method allows comprehensive investigations of the effects of multiple potentially neurotoxic chemicals, and it may facilitate investigations of the mechanisms behind decreases in GluR2 expression.

## Conflict of interest

The authors declare that there are no conflicts of interest.

## Appendix A Supplementary data

Transparency document
